# Association between dietary calcium and phosphorus intakes, dietary calcium/phosphorus ratio and bone mass in the Korean population

**DOI:** 10.1186/1475-2891-13-114

**Published:** 2014-12-13

**Authors:** Kyung-Jin Lee, Kyung-Soo Kim, Ha-Na Kim, Jin-A Seo, Sang-Wook Song

**Affiliations:** Department of Family Medicine, St. Vincent’s Hospital, College of Medicine, The Catholic University of Korea, 93 Jungbudaero, Paldal-gu, Suwon, Gyeonggi-do 442-723 Republic of Korea; Department of Family medicine, Seoul St. Mary’s Hospital, College of Medicine, The Catholic University of Korea, Seoul, South Korea

**Keywords:** Calcium, Phosphorus, Calcium/phosphorus ratio, Bone mineral density, Korean adults

## Abstract

**Background:**

Osteoporosis has become a major public health issue. Among various factors affected bone health, not only dietary calcium and phosphorus intakes, but also the dietary calcium/phosphorus ratio could relate to bone health. Therefore, we evaluated whether dietary calcium and phosphorus intakes, and dietary calcium/phosphorus ratio are associated with bone mass in Korean adults ≥ 20 years of age.

**Methods:**

The analysis used data from the Korean National Health and Nutrition Examination Survey, a cross-sectional survey of Korean civilians, conducted from January to December 2010. A total of 4,935 participants (2,309 men and 2,626 women) were analyzed in this study. Dietary calcium and phosphorus intakes of the participants were estimated using 24-h dietary recall. Bone mass densities for the whole body, femoral neck, and lumbar spine were measured by dual-energy X-ray absorptiometry.

**Results:**

Dietary calcium intake and dietary calcium/phosphorus ratio were positively related to bone mass density for femoral neck in men ≥ 50 years of age (p = 0.046 and 0.041, respectively). Dietary calcium intake showed positive associations with bone mass density for whole body in premenopausal women (p = 0.022).

**Conclusions:**

Increased calcium intake and high dietary calcium/phosphorus ratio might have favorable effects on bone mass in Korean adults. Additional gender- and age-specific studies are needed to further identify the influence of calcium and phosphorus intakes, and the dietary calcium/phosphorus ratio on bone mass.

## Background

Osteoporosis is a skeletal condition characterized by low bone mass and architectural deterioration of bone tissue [1]. Osteoporosis has become one of the public health issues because it is associated with an increased risk of osteoporotic fractures and mortality that contribute to a large socio-economic burden [2–4]. Furthermore, the rates of osteoporosis and osteoporotic fractures are high and have been rapidly increasing worldwide. In the United States in 2010, the prevalence of osteoporosis and low bone mass in adults older than 50 years of age was 10.3% and 43.9% [5]. In addition, the number of osteoporotic fractures exceeds 1.5 million per year, and it has been projected that hip fractures will increase from an estimated 1.7 million in 1990 to 6.3 million in 2050 [6]. The number of hip fractures in the Asia-Pacific area is expected to increase continuously, and is projected to reach 3.25 million by 2050 [7]. In Korea in 2009, the prevalence of osteoporosis in adults older than 50 years of age was 23.1%, and the prevalence in men and women was 8.1% and 38.7%, respectively [8].

Although osteoporosis has been focused primarily on postmenopausal women, osteoporosis in men has become a public health concern. Age-specific incidence rate of hip fracture in men was about half compared with that in women, but the mortality in men with hip fracture was fourfold higher than that in women [9]. Additionally, it is important not to overlook low bone mass in premenopausal women since the occurrences of osteoporosis in the future might be associated with the insufficient acquirement of peak bone mass at a young age [10].

There are numerous factors that influence on bone mass, such as age, body weight, physical activity, cigarette smoking, excessive alcohol intake or concomitant diseases [11]. Among them, the nutrients are also one of the associated components in the achievement of peak bone mass and the control of bone loss. Previous studies have been shown that various nutrients such as vitamin A and its precursors [12, 13], ascorbic acid [13], vitamin K [14, 15], sodium [16], magnesium [17, 18], calcium and vitamin D [19, 20], and phosphorus [21, 22], as well as various foods [23–26] are related to bone mass.

Hydroxylapatite, which is one of the primary mineral compounds in osseous tissue is composed of calcium and phosphate; thus, adequate intake of calcium and phosphorus might be important for bone health [27]. A number of studies have investigated the association between dietary calcium and phosphorus intakes and bone mass [28, 29]. In addition, there were several studies that high dietary calcium/phosphorus ratio might have a positive influence on bone mass [28, 30]. However, to date, no studies in Korean population have focused on the correlation between bone mass and not only dietary calcium and phosphorus intakes but also dietary calcium/phosphorus ratio. Therefore, we evaluated whether dietary calcium and phosphorus intakes and dietary calcium/phosphorus ratio are associated with bone mass in Korean adults classified as men younger or older than 50 years of age and pre- or postmenopausal women using data from the Korean National Health and Nutrition Examination Survey (KNHANES) V-1.

## Methods

### Study population

The present study utilized data collected from the KNHANES V-1, which was conducted between January 2010 and December 2010. The KNHANES assessments are implemented by the Korea Centers for Disease Control and Prevention (KCDC) at 3-year intervals to evaluate the status of public health and to provide baseline data for the development, establishment, and assessment of public health policies in the Korean population. The KNHANES data are obtained from participants who are non-institutionalized, older than 1 year of age, and who were selected using a stratified multi-stage cluster probability sampling design to ensure an independent, homogeneous, and nationally representative sampling. The data consist of household interviews, anthropometric and biochemical measurements, and nutritional status assessments. All protocols were approved by the Institutional Review Board of the KCDC, and the participants provided written informed consent at baseline.

The KNHANES V-1 recruited 10,938 participants, and 8,958 fully completed the survey (participation rate: 81.9%). Of these 8,958 participants, the present cross-sectional study initially examined data of 6,939 adults who were 20 years of age or older regarding bone mass measured by dual-energy X-ray absorptiometry (DXA). Participants missing information or values for major variables (n = 902), with abnormal daily energy intake (< 500 kcal or > 5000 kcal; n = 87), taking hormone replacement therapy (n = 338), with a past history of spine, hip, or wrist fractures (n = 228), taking medicine for osteoporosis (n = 32), and with known renal failure (n = 7), liver cirrhosis (n = 16), thyroid disease (n = 203), rheumatoid arthritis (n = 114), or cancer (n = 77) were excluded. Thus, the final sample of the present study included 4,935 participants. The present study was approved by the Institutional Review Board of the Catholic University of Korea (IRB approval number: VC14EIME0161).

### Dietary assessments

Trained interviewers estimated dietary calcium and phosphorus intakes of the participants using 24-h dietary recall (milligrams per day). Additionally, tools such as food models, two-dimensional food volumes, and containers were also used to assist participants’ recall regarding nutrient intake. A qualitative food frequency questionnaire for 63 common food items that comprised 10 frequency responses was used to obtain additional dietary information.

### Bone mass measurements

Bone mass density (BMD, g/cm^2^) for the whole body, femoral neck, and lumbar spine (1–4) were measured by DXA (DISCOVERY-W fan-beam densitometer, Hologic, Inc., Waltham, Mass., USA). All BMD measurements were performed according to a standardized protocol and guidelines based on the International Society for Clinical Densitometry official positions [31].

### Laboratory measurements

Blood samples were collected from the antecubital vein of each participant after at least 12 h of fasting. The samples were processed, immediately refrigerated, transported in cold storage to the Central Testing Institute in Seoul, Korea, and analyzed within 24 h of arrival at the testing facility. Serum ferritin level was measured by immunoradiometric assay using a γ-counter (1470 Wizard, PerkinElmer, Turku, Finland), and serum alkaline phosphatase was measured using an auto-analyzer (Hitachi Automatic Analyzer 7600, Hitachi, Japan). Serum total 25-hydroxyvitamin D concentrations were measured by radioimmunoassay methods using a γ-counter (1470 Wizard, PerkinElmer, Turku, Finland), and the standard deviation index was 0.50 or less during three times assessment in Vitamin D External Quality Assessment Scheme. The coefficients of variation for intra- and inter-assay were 2.9-5.5 and 6.3-12.9% respectively and the limits of detection were 1.5 ng/mL.

### Clinical and anthropometric measurements

The anthropometric measurements of the participants were conducted by specially trained examiners. Height and weight were measured following an overnight fast while the participants wore a lightweight gown, waist circumference was assessed using a measuring tape in the horizontal plane around the umbilical region after exhaling, and body mass index (BMI) was calculated as a participant’s weight (in kilograms) divided by the square of their height (in meters).

Self-reported information regarding age, gender, smoking, alcohol consumption, residential area, household income, education level, the amount of physical activity, and the presence of dietary supplements and in women, total duration of breast feeding were obtained. Cigarette smoking was classified into three groups based on current use estimates: non-smoker, ex-smoker, and current smoker. Alcohol consumption was classified into three groups: abstinence (no alcoholic drinks consumed within the last year), moderate drinking (less than 14 standard drinks consumed per week for men or seven for women), and heavy drinking (more than 14 standard drinks consumed per week for men or seven for women). Residential area was classified as either urban or rural. Household income was classified using monthly equivalised household income (quartiles), which was estimated as the total monthly household income divided by the square root of the total number of household members. Education level was classified as the period of education (< 10 years and ≥ 10 years). Physical activity was classified as the metabolic equivalent of task minutes per week (MET-minutes per week) and was calculated using the scoring protocol of the Korean version of the International Physical Activity Questionnaire short form: low (< 600 MET-minutes per week), moderate (≥ 600 to < 3,000 MET-minutes per week), or high (≥ 3,000 MET-minutes per week). Participants taking any dietary supplementation for 2 weeks or more during the previous 1 year were defined as ones taking ‘dietary supplement’.

### Statistical analysis

To analyze the data, which were obtained using a complex sampling design, the SAS PROC SURVEY module which considers strata, clusters, and weights was utilized. All analyses were performed using sample weights from the KNHANES V-1. The gender-specific characteristics of the study population were analyzed using independent t-tests for continuous variables and Chi-squared tests for dichotomous variables, and all values were expressed as means **±** standard errors, percentages, or as geometric means and 95% confidence intervals for skewed distributions. Variables with skewed distributions were analyzed after a logarithmic transformation (log-transformation) was performed. The correlations among dietary calcium, dietary phosphorus, the calcium/phosphorus ratio and BMD were analyzed using a Pearson’s correlation analysis and the associations among dietary calcium, dietary phosphorus, and the calcium/phosphorus ratio and BMD were analyzed using a multiple regression analysis. Model 1 was adjusted for age, income, education, residential area, alcohol, smoking, physical activity, energy intake per day, the presence of dietary supplements, and total duration of breast-feeding in women. Model 2 was adjusted for age, income, education, residential area, alcohol, smoking, physical activity, energy intake per day, the presence of dietary supplements, total duration of breast-feeding in women, and BMI. Model 3 was adjusted for age, income, education, residential area, alcohol, smoking, physical activity, energy intake per day, the presence of dietary supplements, total duration of breast-feeding in women, BMI, and alkaline phosphatase, ferritin, and vitamin D levels. All statistical analyses were conducted using the SAS software (ver. 9.2, SAS Institute; Cary, NC, USA), and p values < 0.05 were considered to indicate statistical significance.

## Results

This study was conducted using a total of 4,935 participants (2,309 men and 2,626 women). The mean daily intake of dietary calcium was 588.0 mg for men younger than 50 years of age and 589.8 mg for men 50 years of age or older (p = 0.918), 481.1 mg for premenopausal women and 434.0 mg for postmenopausal women (p <0.001). The mean daily intake of dietary phosphorus was 1435.3 mg for men younger than 50 years of age and 1337.5 mg for men 50 years of age or older (p <0.001), 1079.8 mg for premenopausal women and 968.6 mg for postmenopausal women (p <0.001). The daily calcium/phosphorus ratio was 0.38 for men younger than 50 years of age and 0.40 for men 50 years of age or older (p = 0.005), 0.41 for premenopausal women and 0.39 for postmenopausal women (p = 0.017) (Table 1).

**Table 1:** **Clinical and laboratory characteristics of the study participants**

	Men			Women		
	< 50 years (n = 1,405)	≥ 50 years (n = 904)	p value	Postmenopausal (n = 1,758)	Premenopausal (n = 868)	p value
Age (years)	30.4 ± 0.3	61.3 + 0.4	<0.001	30.5 ± 0.3	62.9 ± 0.5	<0.001
Current smoker (%)	45.8	33.1	<0.001	4.6	5.3	0.663
Heavy drinker (%)	22.2	33.0	<0.001	5.4	2.2	0.001
Residential area (Urban,%)	82.3	67.3	<0.001	83.0	63.9	<0.001
Household income (Q1,%)	12.4	23.2	<0.001	10.8	33.7	<0.001
Education level (≥10 years,%)	75.7	47.2	<0.001	73.2	19.7	<0.001
Physical activity (%)			0.483			0.042
Low	29.6	29.9		39.2	37.9	
Moderate	41.2	38.4		43.0	38.5	
High	29.2	31.7		17.8	23.6	
Dietary supplement (yes,%)	74.7	70.2	0.06	68.7	61.6	0.007
Height (cm)	170.7 ± 0.3	167.2 ± 0.2	<0.001	159.1 ± 0.2	152.7 ± 0.3	<0.001
Weight (kg)	68.8 ± 0.4	66.3 ± 0.4	<0.001	55.8 ± 0.3	56.8 ± 0.3	0.021
Body mass index (kg/m^2^)	23.5 ± 0.1	23.7 ± 0.1	0.264	22.0 ± 0.1	24.3 ± 0.1	<0.001
Waist circumference (cm)	80.6 ± 0.3	85.2 ± 0.4	<0.001	73.0 ± 0.4	82.6 ± 0.3	<0.001
Alkaline phosphatase (IU/L)^*^	268.9(260.0-278.1)	231.6(226.5-236.9)	<0.001	207.0(202.1-212.0)	250.4(245.3-255.6)	<0.001
Ferritin (ng/mL)^*^	81.8(77.8-85.9)	92.5(85.8-99.7)	0.007	22.2(21.0-23.4)	55.2(51.2-59.5)	<0.001
Serum 25(OH)D (ng/mL)	17.7 ± 0.3	20.9 ± 0.4	<0.001	15.8 ± 0.3	18.3 ± 0.4	<0.001
Calcium intake (mg/day)	588.0 ± 11.6	589.8 ± 14.1	0.918	481.1 ± 8.7	434.0 ± 11.7	<0.001
Phosphorus intake (mg/day)	1435.3 ± 20.0	1337.5 ± 19.9	<0.001	1079.8 ± 12.9	968.6 ± 20.1	<0.001
Calcium/phosphorus ratio^*^	0.38(0.37-0.39)	0.40(0.39-0.41)	0.005	0.41(0.40-0.42)	0.39(0.37-0.41)	0.017
Bone mass density (g/cm^2^)						
Whole body	1.16 ± 0.01	1.16 ± 0.01	0.861	1.10 ± 0.01	1.00 ± 0.01	<0.001
Lumbar	0.94 ± 0.01	0.96 ± 0.01	0.196	0.95 ± 0.01	0.81 ± 0.01	<0.001
Femoral neck	0.85 ± 0.01	0.76 ± 0.01	<0.001	0.76 ± 0.01	0.63 ± 0.01	<0.001

Values are expressed as means ± standard errors or percentages and as geometric means (95% confidence intervals) for skewed distributions*. Variables with skewed distributions performed log-transformation. *Q1* Quartile 1(the lowest household income), *25*(*OH)D* 25-hydroxyvitamin D.

The correlation coefficients for dietary calcium and phosphorus intake, and dietary calcium/phosphorus ratio with bone mass are shown in Table 2. Significant positive correlations were observed between dietary calcium intake and dietary calcium/phosphorus ratio and BMD for all measured regions, and dietary phosphorus intake and BMD for whole body and femoral neck regions in men 50 years of age and older. In men younger than 50 years of age, dietary calcium and dietary calcium/phosphorus ratio were related to lumbar spine BMD positively and to whole body BMD negatively. In postmenopausal women, there were positive relationships between dietary calcium and phosphorus intakes, dietary calcium/phosphorus ratio, and BMD for lumbar spine and femoral neck and in premenopausal women, dietary calcium/phosphorus ratio showed a positive association with femoral neck BMD.

**Table 2:** **Correlations between dietary calcium, phosphorus, calcium/phosphorus ratio and bone mass density**

	Correlation coefficient (r) with bone mass density		
	Whole body	Lumbar	Femoral neck
Men			
< 50 years			
Calcium intake	-**0.073** ^*^	**0.102** ^**^	0.015
Phosphorus intake	**-**0.050	**0.129** ^**^	0.050
Ca/P ratio	-**0.070** ^*^	**0.134** ^**^	0.043
≥ 50 years			
Calcium intake	**0.147** ^**^	**0.171** ^**^	**0.180** ^**^
Phosphorus intake	**0.121** ^**^	0.072	**0.133** ^**^
Ca/P ratio	**0.110** ^**^	**0.101** ^**^	**0.126** ^**^
Women			
Premenopausal			
Calcium intake	-0.001	-0.001	0.004
Phosphorus intake	0.015	0.029	0.044
Ca/P ratio	0.036	0.048	**0.068** ^*^
Postmenopausal			
Calcium intake	0.064	**0.224** ^**^	**0.177** ^**^
Phosphorus intake	0.081	**0.148** ^**^	**0.144** ^**^
Ca/P ratio	0.069	**0.151** ^**^	**0.118** ^**^

*p < 0.05, **p <0.01. *Ca/P ratio* Calcium/phosphorus intake ratio. Results in bold indicate statistical significance at the 0.05 level.

The multivariate-adjusted associations between dietary calcium and phosphorus intakes, calcium/phosphorus intake ratio and BMD are shown in Tables 3 and 4. After adjusting for confounding factors such as age, income, education, residential area, alcohol, smoking, physical activity, energy intake per day, the presence of dietary supplements, total duration of breast-feeding in women, BMI, serum alkaline phosphatase, ferritin, and vitamin D levels, dietary calcium intake and dietary calcium/phosphorus ratio were positively related to BMD in the femoral neck of men older than 50 years of age and in premenopausal women, dietary calcium intake showed positive associations with the whole body BMD.

**Table 3:** **Multivariate-adjusted associations between dietary calcium, phosphorus, calcium/phosphorus ratio and bone mass density among men**

	<50 years						≥ 50 years					
	Whole body		Lumbar		Femoral neck		Whole body		Lumbar		Femoral neck	
	ß	p	ß	p	ß	p	ß	p	ß	p	ß	p
Model 1												
Calcium intake	0.000005	0.648	0.000009	0.546	0.000007	0.552	**0.000034**	**0.005**	**0.000050**	**0.014**	**0.000039**	**0.002**
Phosphorus intake	0.000012	0.109	0.000015	0.092	0.000011	0.197	0.000014	0.118	0.000012	0.333	0.000015	0.075
Ca/P ratio	-0.018726	0.105	-0.008208	0.499	-0.014333	0.327	**0.026552**	**0.019**	**0.039903**	**0.016**	**0.033198**	**0.001**
Model 2												
Calcium intake	0.000001	0.888	0.000004	0.764	0.000002	0.823	**0.000026**	**0.021**	0.000034	0.192	**0.000029**	**0.014**
Phosphorus intake	0.000012	0.099	0.000015	0.080	0.000011	0.162	0.000013	0.212	0.000006	0.629	0.000011	0.174
Ca/P ratio	**-0.022948**	**0.045**	-0.013631	0.265	-0.020154	0.160	0.020238	0.069	0.027653	0.110	**0.025128**	**0.013**
Model 3												
Calcium intake	0.000002	0.804	0.000006	0.650	0.000003	0.741	0.000022	0.065	0.000026	0.191	**0.000023**	**0.046**
Phosphorus intake	0.000013	0.089	0.000016	0.073	0.000014	0.086	0.000008	0.359	0.000001	0.937	0.000009	0.300
Ca/P ratio	-0.020239	0.094	-0.012013	0.335	-0.018413	0.216	0.017843	0.125	0.023537	0.179	**0.020974**	**0.041**

Model 1: adjustment for age, household income, education level, residential areas, alcohol consumption, smoking, physical activity, energy intake per day, and dietary supplement. Model 2: adjustment for model 1 + body mass index. Model 3: adjustment for model 2 + alkaline phosphatase, ferritin, and 25-hydroxyvitamin D. *Ca/P ratio* Calcium/phosphorus intake ratio. Results in bold indicate statistical significance at the 0.05 level.

**Table 4:** **Multivariate-adjusted associations between dietary calcium, phosphorus, calcium/phosphorus ratio and bone mass density among women**

	Premenopausal						Postmenopausal					
	Whole body		Lumbar		Femoral neck		Whole body		Lumbar		Femoral neck	
	ß	p	ß	p	ß	p	ß	p	ß	p	ß	p
Model 1												
Calcium intake	0.000018	0.592	0.000007	0.491	0.000004	0.696	0.000013	0.270	0.000031	0.064	0.000019	0.148
Phosphorus intake	0.000010	0.149	0.000003	0.643	0.000001	0.882	0.000006	0.486	0.000014	0.371	0.000014	0.187
Ca/P ratio	0.008565	0.313	0.000873	0.922	0.001513	0.871	0.007024	0.412	0.010293	0.402	-0.002908	0.751
Model 2												
Calcium intake	**0.000020**	**0.029**	0.000009	0.294	0.000006	0.501	0.000013	0.286	0.000031	0.052	0.000018	0.147
Phosphorus intake	0.000011	0.097	0.000005	0.486	0.000001	0.777	0.000003	0.698	0.000008	0.583	0.000009	0.322
Ca/P ratio	0.009746	0.224	0.003217	0.701	0.003813	0.632	0.009180	0.298	0.013955	0.233	0.000190	0.982
Model 3												
Calcium intake	**0.000022**	**0.022**	0.000012	0.169	0.000005	0.564	0.000009	0.454	0.000025	0.112	0.000012	0.300
Phosphorus intake	0.000012	0.072	0.000007	0.294	0.000002	0.725	0.000002	0.765	0.000007	0.608	0.000008	0.385
Ca/P ratio	0.008914	0.269	0.002167	0.798	0.001785	0.827	0.005130	0.596	0.006959	0.549	-0.005030	0.534

Model 1: adjustment for age, household income, education level, residential areas, alcohol consumption, smoking, physical activity, energy intake per day, dietary supplement, and duration of breast feeding. Model 2: adjustment for model 1 + body mass index. Model 3: adjustment for model 2 + alkaline phosphatase, ferritin, and 25-hydroxyvitamin D. *Ca/P ratio* Calcium/phosphorus intake ratio. Results in bold indicate statistical significance at the 0.05 level.

## Discussion

The goal of this study was to evaluate the relationships between dietary calcium, phosphorus, calcium/phosphorus intake ratio, and bone mass in Korean adults. There were positive relationships between dietary calcium intake, calcium/phosphorus intake ratio and femoral neck BMD in men older than 50 years, and dietary calcium intake showed positive associations with BMD for whole body in premenopausal women.

The association between dietary calcium and phosphorus intake, and bone health has been addressed by a number of experimental and epidemiological studies. In a cross-sectional study conducted in Chinese freshmen, adequate calcium intake was a negative association with the risk of osteopenia/osteoporosis [32]. In experimental studies in rats, BMD in a group fed a high-phosphate diet decreased significantly than that in the control group [33, 34]. In a randomized controlled trial conducted on healthy women, high phosphorous intake adversely influenced bone metabolism by increasing bone resorption and decreasing bone formation [35]. Along the same lines as other studies on calcium intake and bone mass, as the results of the multivariate-adjusted analysis in this study, dietary calcium intake was positively related to BMD for the femoral neck of men 50 years of age and older, and for the whole body of premenopausal women. However, dietary phosphorous intake showed no association with bone mass in all groups of this study.

Dietary phosphorus is contained in not only protein-rich foods such as meat, dairy products, poultry and fish, but also processed foods that contain phosphate-based additives [36]. In fact, phosphorus is a material for bone formation [27], but excessive phosphorus intakes elevate serum phosphorus levels and disturb the hormonal regulation of calcium and phosphorus, which may result in decreased bone strength and an increased risk of fractures [37], and the deleterious effects of excessive phosphorus intake on bone increase when calcium intake is low [38]. Therefore, adequate dietary calcium intake is needed to overcome the harmful effects of a high phosphorus intake on bone health [39]. In Korea, calcium intake is lower than the dietary reference intakes for Korean [8], and calcium intake in much of the developing countries including Korea is lower than that in the developed countries [8, 40]. In addition to the low contents of calcium in the Korean diet, many dietary factors unique to traditional Korean foods can also influence the efficiency of calcium absorption. The traditional Korean diet containing high intake of rice, kimchi, and vegetables is composed of low lipids and proteins, and high carbohydrates, and is high in substances such as oxalate, phytate, and fiber that could hamper the absorption of calcium. Additionally, in western countries, excess intake of phosphorus has been an issue on bone health [21, 41, 42], but in Korea, there have been few concerns on high intake of phosphorus, especially the intakes from food additives. Furthermore, in many countries including Korea, dietary phosphorus intake is higher than recommended levels [41] whereas dietary calcium intake is lower than the nutritional recommendations [43, 44], therefore dietary calcium/phosphorus ratio is low [45, 46]. In this study, dietary calcium/phosphorus ratio was positively related to BMD for the femoral neck in men 50 years of age and older. The low dietary calcium/phosphorus ratio might alter the homoeostasis of calcium metabolism and be associated with increased serum parathyroid hormone concentration, which may cause increased bone resorption [47, 48], suggesting that high calcium/phosphorus intake ratio might be needed to the optimal bone health.

In a cross-sectional study for determining the predictors of bones mass in healthy older men, body composition factors, such as body weight or lean mass were the main factors of bone mass prediction [49], and in another cross-sectional study conducted in adult men, when calcium intake is adequate, protein intake, lean body mass as well as phosphorus intake were beneficial for maintaining bone mass [50]. Thus, some factors including protein intake, body weight or lean mass could influence on the association between phosphorus intake and bone mass. In this study, positive correlations were observed between dietary phosphorus intake and BMD for femoral neck and whole body in men older than 50 years and older and for lumbar spine in men younger than 50 years, but after adjusting for covariates including daily energy intake, BMI, and serum vitamin D levels, the correlations disappeared. Therefore, when evaluating the associations between dietary phosphorus intake and bone mass, the covariates that have effects on the relationship of phosphorus intake and bone mass should be carefully considered.

A number of studies investigating dietary calcium, phosphorus, calcium/phosphorus intake ratio, and osteoporosis have been conducted in women. Ito et al. [28] reported that dietary calcium intake and dietary calcium/phosphorus ratio were positively associated with the bone mass in radius of women aged 18-22 years, and Brot et al. [30] found that dietary calcium/phosphorus ratio was positively related to whole-body mineral content and bone mass in the spine and femoral neck in women aged 45-58 years. However, in the present study, there were only positive associations between dietary calcium intake and whole body BMD in premenopausal women. The results may be due to other factors affected bone mass, such as thyroid hormone or estrogen [51, 52]. However, the possible factors were not considered in the present study because the factors were not measured in the KNHANES, therefore, additional studies adjusting such covariates are warranted to clarify the associations between dietary calcium and phosphorus intakes, calcium/phosphorus intake ratio and bone mass in Korean women.

The present study has several limitations. First, this study was conducted using a cross-sectional design. Second, data about the dietary calcium and phosphorus intake were collected via a 24 h dietary recall by the participants, which could have resulted in a recall bias. A 1-day 24-h recall could not be enough to estimate the typical daily intake of an individual due to the day-to-day variability in food and nutrient intakes [53]. Third, we did not consider other nutritional factors such as dietary sodium or vitamin K that might affect bone health or mass, since the factors were not measured in the KNHANES. However, the strengths of this study were that the data were collected using a representative nationwide survey of the South Korean population and that this is the first study of Korean adults to investigate gender- and age-specific associations between dietary calcium and phosphorus intakes, and dietary calcium/phosphorus ratio and bone mass.

In conclusion, dietary calcium intake and calcium/phosphorus ratio in men older than 50 years of age, and dietary calcium intake in premenopausal women had small but significant positive associations with bone mass in femoral neck or whole body. These findings suggest that increased calcium intake and high calcium/phosphorus intake ratio might have beneficial effects on bone mass in Korean population. Further gender-and age-specific studies are needed to further identify the influence of calcium and phosphorus intakes, and the dietary calcium/phosphorus ratio on bone mass, and to evaluate the adequate amounts of dietary calcium and phosphorus intakes on bone health.

## Authors’ information

KJL: M.D., Department of Family Medicine, St. Vincent’s Hospital, College of Medicine, The Catholic University of Korea.

KSK: M.D., Ph.D., Professor, Department of Family medicine, Seoul St. Mary’s Hospital, College of Medicine, The Catholic University of Korea.

HNK: M.D., MSc., Clinical assistant professor, Department of Family Medicine, St. Vincent’s Hospital, College of Medicine, The Catholic University of Korea.

JAS: M.D., Department of Family Medicine, St. Vincent’s Hospital, College of Medicine, The Catholic University of Korea.

SWS: M.D., Ph.D., Professor, Department of Family Medicine, St. Vincent’s Hospital, College of Medicine, The Catholic University of Korea.

## Abbreviations

BMD: Bone mineral density
BMI: Body mass index
DXA: Dual-energy X-ray absorptiometry
KCDC: The Korea Centers for Disease Control and Prevention
KNHANES: Korean National Health and Nutrition Examination Survey
MET: Metabolic equivalent of task.
